# FIGO 2018 Versus Ontogenetic Staging for Locally Advanced Cervical Cancer: An International Multicenter Cohort Study Comparing the Two Classifications and Their Prognostic Implications

**DOI:** 10.3390/cancers18040689

**Published:** 2026-02-19

**Authors:** Bruno Rezende, Benjamin Wolf, Vinicius Colman, Rivadavio de Oliveira, Svetlana Kulikova, Pavel Sorokin

**Affiliations:** 1 Department of Gynecologic Oncology, Londrina Cancer Hospital, Lucilla Ballalai, 212-Jardim Petrópolis, Londrina 86015-520, Brazil; 2 Department of Gynecology, University Hospital Leipzig, Liebigstrasse 18, 04103 Leipzig, Germany; benjamin.wolf@medizin.uni-leipzig.de; 3 Edwin L. Steele Laboratories, Department of Radiation Oncology, Massachusetts General Hospital and Harvard Medical School, 100 Blossom St, Cox-734, Boston, MA 02114, USA; 4 Department of Surgical Oncology, Londrina Cancer Hospital, Lucilla Ballalai, 212-Jardim Petrópolis, Londrina 86015-520, Brazil; viniciuspereiracolman@hotmail.com; 5 Department of Clinical Oncology, Londrina Cancer Hospital, Lucilla Ballalai, 212-Jardim Petrópolis, Londrina 86015-520, Brazil; riva-mt@uol.com.br; 6 Department of Gynecologic Oncology, Moscow City Oncology Hospital No62, Istra 27, 143423 Moscow, Russia; s_s76@mail.ru (S.K.); sor-pavel@ya.ru (P.S.)

**Keywords:** FIGO 2018, ontogenetic staging, cervical cancer

## Abstract

Background/Objective: The aim of this study was to compare two different staging systems (FIGO 2018 and ontogenetic staging) for cervical cancer in a locally advanced setting and to determine which one provides better prognostic stratification and a stronger association with oncological outcomes. Methods: A multicenter retrospective cohort study was conducted in patients with cervical cancer (FIGO 2018 stages IIB-IVA) primarily treated with chemoradiotherapy. The primary endpoint was the difference in accuracy for predicting cancer-specific survival at 3 years between the ontogenetic tumor staging system and the FIGO 2018 staging system. Secondary endpoints assessed the same difference in prognostic power with respect to recurrence-free survival and overall survival. Results: Of 341 patients with locally advanced cervical cancer, the ontogenetic tumor staging system demonstrated superior prognostic performance compared to the FIGO 2018 system. The ontogenetic system showed clear separation of Kaplan–Meier curves for recurrence-free, overall, and cancer-specific survival, unlike the overlapping FIGO 2018 survival curves. Multivariable Cox models controlling clinicopathological factors revealed a progressive, significant worsening of cancer-specific survival across ontogenetic tumor (oT) stages, which was not observed with FIGO staging. Formal comparisons using Harrell’s C-index, time-dependent areas under the curve (AUC), and Akaike’s information criterion confirmed the ontogenetic model’s significantly better discriminative ability and fit. Furthermore, ontogenetic staging provided significant risk stratification within the FIGO IIIC nodal-positive group and among patients with parametrial invasion, demonstrating its refined prognostic utility. Conclusions: Ontogenetic tumor staging can be effectively applied using imaging alone and was superior to the FIGO 2018 classification in this study.

## Simple Summary

This study looked at a new way of classifying how cervical cancer spreads in the body, the ontogenetic tumor (oT) staging system. Unlike the traditional FIGO staging system, this new method is based on a more detailed anatomic understanding of how tumors grow and move, as seen on modern medical scans. This is the first time this oT staging approach has been successfully applied using imaging tests alone in advanced cervical cancer. Our results show that this new system works better than the current FIGO 2018 system in several important ways. These findings suggest that we may need to rethink how we classify cervical cancer. Because this new method is more accurate and better at predicting patient outcomes, it could augment existing staging strategies to improve cervical cancer management in the future.

## 1. Introduction

Cervical cancer represents the fourth leading cause of cancer death in women, with an estimated 348,189 deaths worldwide in 2022. In high-income countries, there has been a decrease in the incidence of invasive disease. However, many patients are still diagnosed at an advanced stage, especially in lower-resource countries [1]. The optimal treatment and follow-up strategy is often dictated by disease stage; therefore, understanding the distribution of stages and their correlation with clinical outcomes is critical. The revised 2018 International Federation of Gynecology and Obstetrics (FIGO) staging system for cervical cancer [2,3,4], currently the most widely used, relies heavily on imaging for advanced cases and presents several limitations. First, it groups heterogeneous conditions within the same stages—for example, combining proximal paracervical involvement with bladder muscle infiltration in stage IIB and failing to adequately stratify cases in stage IIIC. Second, prognostic outcomes for stage IIIB are notably worse than most stage IIIC1 cases, suggesting inconsistencies in risk stratification [5]. Third, key anatomical definitions, such as those for the parametrium and pelvic sidewall, lack precision, potentially leading to clinical ambiguity. Finally, the FIGO staging system omits certain anatomical regions, including the muscular wall of the bladder and mesorectum, despite evidence that their involvement adversely affects prognosis. Several of these flaws are based on the assumption that cervical cancer spreads isotropically into surrounding tissues along the paths of the least mechanical resistance. However, it has been shown that cervical carcinomas do not spread randomly but rather disseminate selectively within the pelvis and perineum along specific developmental fields defined by their embryological kinship. Based on these observations, Michael Höckel has put forward a novel theory of locoregional cancer progression, the ontogenetic model. This concept suggests that cancer spreads within anatomical compartments determined by the ontogenesis of the normal tissue from which it originated. Malignant progression is inversely related to the fate progression of the normal cell type regarding colonization potential [6,7,8]. As each anatomical structure within the pelvis can be traced to its ontogenetic origin, specific domains defined by ontogenetic anatomy can be delineated and attributed to predictable risks of tumor infiltration. The ontogenetic tumor (oT) staging system is based on these ontogenetic–anatomic domains and offers a detailed consideration of all pelvic anatomical regions susceptible to tumor involvement, each of which has a direct impact on prognosis. This anatomical precision accounts for common imaging findings in locally advanced tumors, a feature not captured by traditional staging systems, such as FIGO. Thus, an argument can be made for basing local cancer staging and therapeutic approaches on ontogenetic rather than solely on traditional anatomical concepts. Although prior publications have demonstrated the prognostic accuracy of the ontogenetic staging system for cervical and vulvar cancer, its clinical utility is limited by its reliance on anatomopathological findings [9,10]. This is a particular drawback for locally advanced tumors, which are conventionally staged by imaging and treated without surgery. It is not yet established whether ontogenetic staging, as assessed non-invasively via imaging, offers advantages over the standard FIGO 2018 classification for locally advanced cervical cancer. To date, no study has compared both staging systems (estimated solely by imaging) in this population. Therefore, the objective of this study was to assess whether the ontogenetic staging system provides additional prognostic power when compared to the conventional FIGO 2018 classification for cervical cancer.

## 2. Methods

### 2.1. Patient and Data Selection

This multicenter retrospective cohort study included patients with locally advanced cervical cancer (FIGO 2018 stages IIB–IVA) that were treated primarily with radiotherapy-based regimens at four centers in Brazil (cities: Londrina, Maringá, Arapongas and Apucarana) and one center in Moscow, Russia, between Feb 2018 and July 2025. The study was approved by the local ethics committee in Brazil (CAAE: 90880125.8.0000.5696) and IRB in Russia. Patients were eligible for inclusion if they had FIGO (2018) stage IIB–IVA cervical cancer, classified as either squamous cell carcinoma, adenocarcinoma, or adenosquamous carcinoma and treated exclusively with concomitant chemoradiotherapy (CCRT), radiotherapy alone, induction chemotherapy + CCRT (same as the INTERLACE study) or pembrolizumab + CCRT (same as the Keynote-A18 study regimen). All participants were required to have pre-treatment pelvic magnetic resonance imaging (MRI) and chest/abdomen computed tomography (CT) scans available for review to be included in the initial analysis.

The radiotherapy techniques used at all centers followed current international guidelines (NCCN), except for the consistent use of intensity-modulated radiotherapy (IMRT), which was not available at all institutions. Patients with lymph node-positive disease were treated according to the same guidelines and received either a radiotherapy boost or extended-field irradiation when indicated.

Exclusion criteria were histological subtypes other than squamous cell carcinoma, adenocarcinoma, or adenosquamous; unavailable images for review (pelvic MRI or abdomen/chest CT scans); concurrent cancers; incomplete relapse information; cases initially treated by surgery; and loss to follow-up after treatment. The staging method was the same in all centers: assessment of local tumor extent with pelvic MRI and retroperitoneum, upper abdomen and chest, which was assessed with CT scans or Positron Emission Tomography/Computed Tomography (PET/CT), if available.

Follow-up adhered to institutional protocols, consistent across all centers: pelvic MRI at three and six months post-treatment; PET/CT was also requested, if available. Physical examinations and symptom counseling were conducted every three months for the first two years, then every six months until five years post-treatment. Additional exams were performed only if new symptoms arose. Recurrence or disease progression was defined based on imaging and RECIST version 1.1 criteria [11]. Equivocal cases underwent image-guided biopsy or repeat imaging after three months.

All data were collected from institutional electronic medical records and radiology department databases.

### 2.2. Imaging and Staging

**Figure 1:**
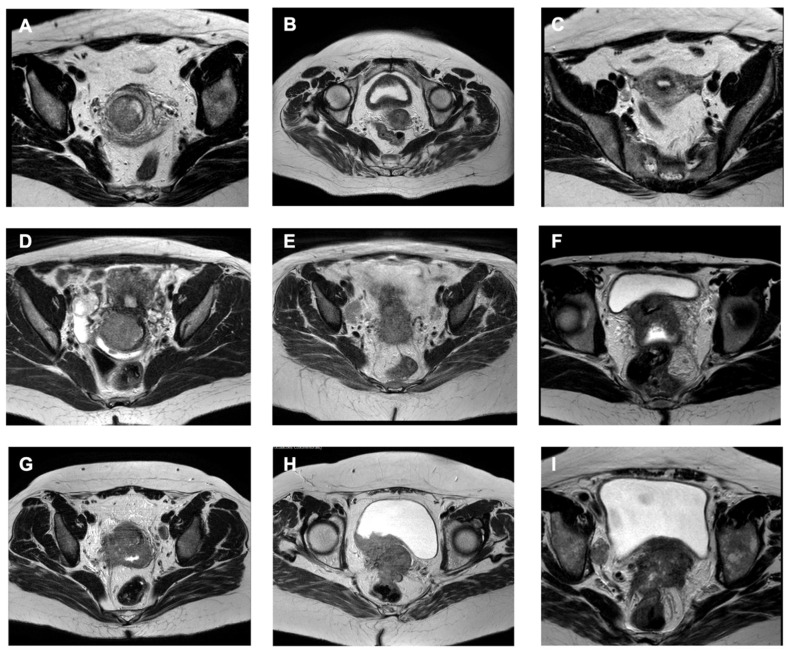
(**A**) Cervical ring stroma disruption without involvement of surrounding fatty tissues (oT2, FIGO IIB); (**B**,**C**) cervical ring stroma disruption without involvement of surrounding fatty tissues (B, oT2) and lymph node metastasis on right side (C, FIGO IIIC1); (**D**) cervical ring stroma disruption without involvement of surrounding fatty tissues (oT2) with lymph node metastasis on right side (FIGO IIIC1); (**E**) suspected involvement of proximal urogenital mesentery on right side (oT3a) and lymph node metastasis (FIGO IIIC1); (**F**) tumor invading bladder muscle and right proximal urogenital mesentery (oT3b, FIGO IIB); (**G**) tumor invading the distal part of urogenital mesentery on right side (oT3b) and lymph node metastasis on left side (FIGO IIIC1); (**H**) invasion of the bladder muscle, bladder mucosa (confirmed by cystoscopy with biopsy) and mesorectum (oT4, FIGO IVa); (**I**) bladder muscle, distal right urogenital mesentery and mesorectum involvement (oT4), and lymph node metastasis on right side (FIGO IIIC1). oT: ontogenetic tumor.

A complete review of all imaging films was performed by the authors and blinded institutional radiologists (blinded to all oncological outcomes). The ontogenetic tumor stage was estimated according to criteria and nomenclature previously proposed [10,12,13,14,15,16,17,18,19,20,21,22,23,24,25] (Table 1 and Table 2). These criteria permit precise anatomical mapping of local tumor spread within the pelvis. Regional and distant lymph node metastases were assessed along the commonly accepted lymphatic drainage regions in the pelvis and the para-aortic region. Invasion of an anatomical area was only considered positive if the imaging was unequivocal and free of artifacts. Detailed regions of tumor involvement were recorded for each patient. Cases in which the exact ontogenetic tumor stage was unclear after the first assessment had their entire pelvic MRI films shared between the authors and radiologists from different centers for joint review. The definitive ontogenetic tumor stage was given only after a unanimous consensus was reached between both parties. FIGO stage IVA required confirmation via cystoscopy or recto sigmoidoscopy with biopsy. Exemplary pelvic MRI images depicting tumor infiltration of various ontogenetic anatomical structures are shown in Figure 1.

Retroperitoneal lymph node evaluation and distant disease were assessed using abdominal and chest CT or PET/CT (SUV max cut-off: 3.85), if available. Positive lymph node status was defined as a short-axis diameter, >1 cm (oval shape) or >0.8 cm (round shape), or the presence of necrosis [16,17] for both pelvic MRI and chest/abdominal CT.

### 2.3. Endpoints and Statistical Analysis

The primary endpoint was the difference in the accuracy of prognosticating cancer-specific survival after 3 years between the ontogenetic and the FIGO staging systems. Secondary endpoints had the same difference in prognostic power with respect to recurrence-free and overall survival. Additional exploratory subgroup analyses were conducted to assess the prognostic relevance of lymph node status and parametrial infiltration.

Relapse-free survival was calculated from the start of treatment until first relapse, diagnosis of persistent disease, or death from any cause. Overall survival was calculated from cancer diagnosis to death from any cause. Cancer-specific survival was calculated from diagnosis to death from the disease.

All statistical analyses were performed using R [18] and R-Studio [19]. Baseline categorical characteristics are reported as absolute numbers and percentages; quantitative data are presented as the median and interquartile range. Statistical significance was set at *p*-value ≤ 0.05.

Kaplan–Meier estimates for three-year survival were computed using the survival package for R and plotted using the ggplot2 [20] and survminer packages [21]. Survival curves for each tumor stage in both classifications (FIGO 2018 and ontogenetic) were compared using the log-rank test. Hazard ratios (HRs) were estimated using univariable and multivariable Cox proportional hazard models via the built-in coxph() function in R, and Wald-type confidence intervals were calculated.

To compare the prognostic discrimination of the two staging systems for cancer-specific survival, we fitted separate multivariable Cox proportional hazard models with each staging system (FIGO and ontogenetic) as the primary covariate. Akaike’s information criterion was calculated for each model, with lower values indicating a better balance between the number of variables included and predictive power.

To investigate the relevance of ontogenetic and FIGO staging, univariable models were built for each staging system. Discrimination was then quantified using Harrell’s concordance index, which was calculated using the Hmisc package for R [22]. To compare the two models formally, we calculated the difference in concordance index and estimated 95% confidence intervals using nonparametric bootstrap resampling with 1000 iterations utilizing the boot package for R [23]. In each bootstrap sample, the two Cox models were refitted, the concordance index was recalculated, and the difference (ontogenetic model–FIGO model) was determined. The distribution of these bootstrap differences was then used to derive bias-corrected and accelerated confidence intervals (CIs) and two-sided *p*-values. A difference in concordance index whose 95% CI did not include zero was considered statistically significant, indicating superior discrimination of one staging system over the other.

As an additional comparison between the staging models, we fitted time-dependent areas under the receiver operating characteristic curves. These areas were estimated using the timeROC framework for right-censored survival data [24]. For each Cox model, we derived individual linear predictors and evaluated discriminatory performance over time. To obtain 95% confidence intervals around the time-dependent areas under the receiver operating characteristic curve estimates, we applied nonparametric bootstrap resampling with replacement (1000 iterations) and calculated percentile-based CIs at each timepoint.

Finally, calibration of the two Cox proportional hazard models was assessed using the riskRegression package in R [25]. Predicted survival probabilities at a fixed horizon (36 months) were obtained with the *predictRisk()* function, and calibration was evaluated with the Score framework using bootstrap resampling (1000 iterations) to reduce overfitting. Calibration plots were generated with plotCalibration().

## 3. Results

A total of 461 patients with locally advanced cervical cancer (FIGO 2018 stage IIB to IVA), treated between 20 February 2018 and 30 July 2025, were identified from four Brazilian centers and one center in Russia. After excluding 120 patients because of primary surgical treatment (60), non-eligible histology (9), unavailable imaging for review (pelvic MRI or CT scans) (47), and loss to follow-up (4), a total of 341 patients were included in the final analysis. Reasons for primary surgical treatment were patient’s choice in cases of early-stage IIb (oT2) tumors amenable to treatment with total mesometrial resection (TMMR), tumors confined to the cervix with a suspicious lymph node on imaging, FIGO IIIC (routinely treated with TMMR in two centers) cases, and FIGO IVA cases associated with fistulas. The median follow-up time was 30 months (interquartile range: 17–40). Clinical and pathological characteristics of the study population are summarized in Table 3.

At the last follow-up, 203 patients remained alive without relapse, 28 were alive following relapse, and 101 had died from the disease after relapsing. An additional 9 patients died from non-cancer-related causes. Patterns of relapse, treatments administered at relapse, and other causes of death are detailed in Tables S1 and S2.

**Figure 2:**
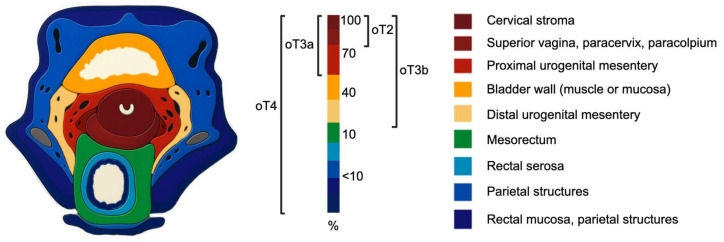
Heat map illustrating the probability of tumor presence across different ontogenetic regions on axial T2-weighted pelvic MRI. The infiltration probability recapitulates ontogenetic tumor stages.

The most common anatomical sites of tumor involvement that were decisive for ontogenetic staging were: the paracervix (without parametrial fatty tissue involvement; 98.9%), superior vagina (82.2%), paracolpium (71%), proximal urogenital mesentery (66.6%), bladder muscle (39.6%), distal urogenital mesentery (30.1%), mesorectum (14.8%), rectal serosa (5.5%), bladder mucosa (4.5%), parietal structures (2.5%) and rectal mucosa (1.1%). Approximately 100 pelvic MRI scans with initial parametrial invasion were shared between the Brazilian and Russian center for review and distinction between oT2 vs. oT3a stages. The specific anatomical areas defining each ontogenetic tumor stage are detailed in Table S3. Figure 2 presents a schematic heat map illustrating the probability of tumor presence across different ontogenetic regions on axial T2-weighted pelvic MRI.

**Figure 3:**
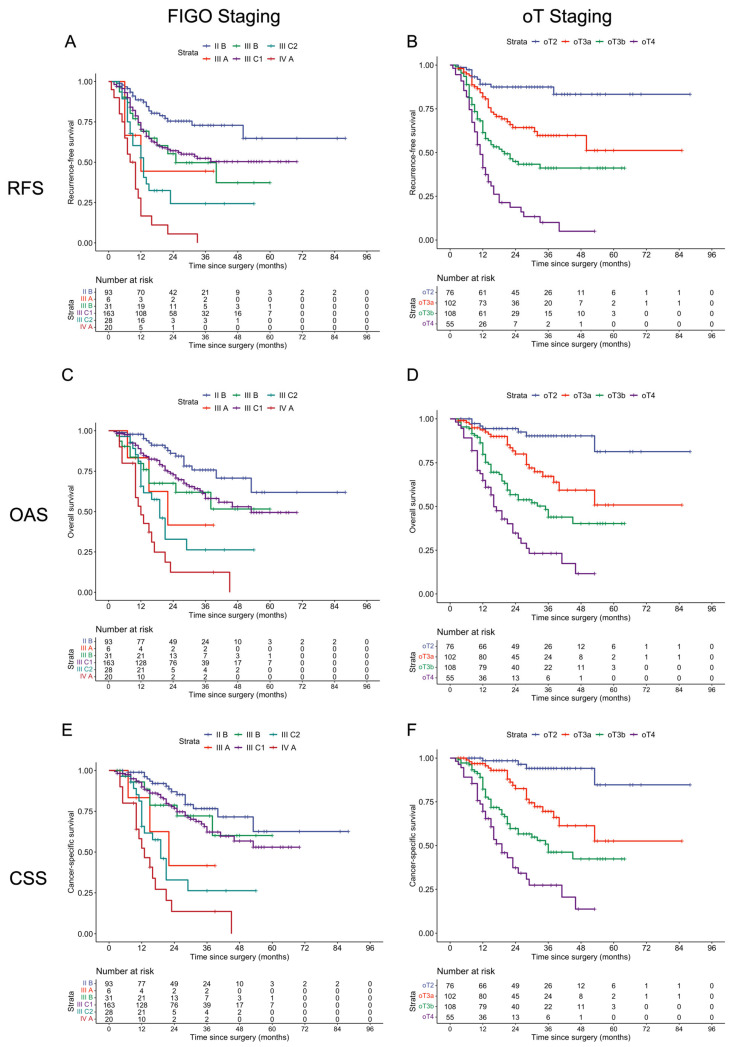
Kaplan–Meier curves showing the probabilities of RFS, OAS, and CSS for FIGO (**A**,**C**,**E**) and oT (**B**,**D**,**F**) staging. Less overlapping and more diverging curves for oT staging indicate better prognostic discrimination. RFS: recurrence-free survival, OAS: overall survival, CSS: cancer-specific survival. FIGO: International Federation of Gynecology and Obstetrics. oT: ontogenetic tumor staging.

The three-year relapse-free survival, overall survival, and cancer-specific survival rates for the entire cohort were 51.7% (46.0–58.2%), 57.7% (51.5–64.5%), and 60.8% (54.5–67.8%), respectively. Kaplan–Meier curves, stratified by subgroups within each staging system, are presented in Figure 3; the corresponding numeric 36-month survival data are summarized in Table S4. Overall, we observed almost no overlap between survival curves of the various ontogenetic stages, while there was substantial overlap and crossing of the FIGO-stage Kaplan–Meier curves (Figure 3).

**Figure 4:**
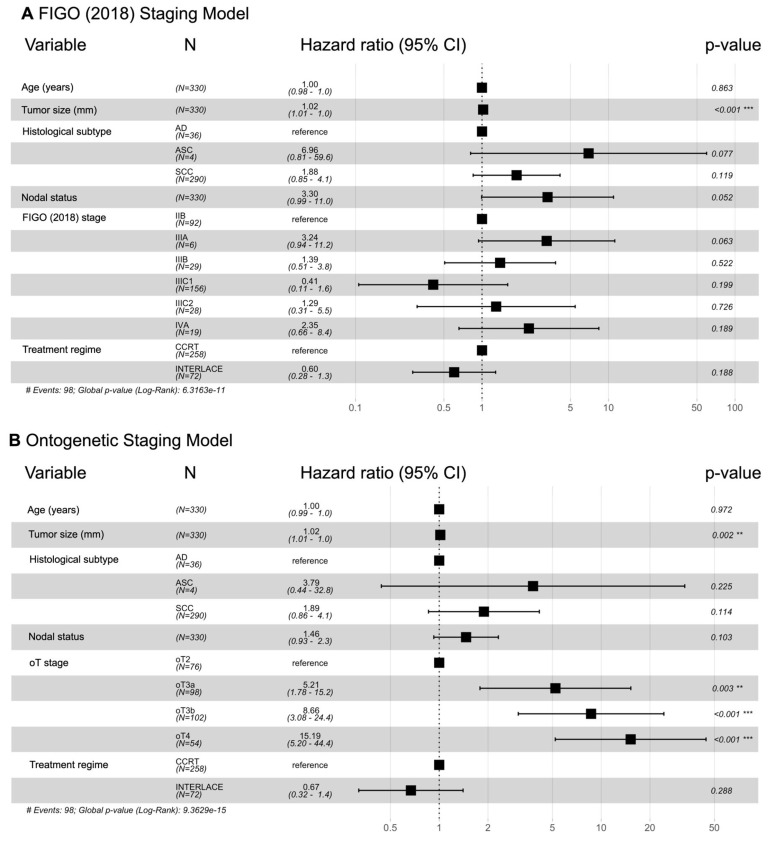
Forest plots depicting hazard ratios (HRs, black squares) with 95% confidence intervals (bracketed lines) of multivariable models including either the 2018 FIGO stage (**A**) or the oT stage (**B**) as the primary variable. Both models control for age, tumor size, histological subtype, and nodal status. HRs for the linear variables age and tumor size are given per year and per millimeter (mm), respectively. Each incremental oT stage differs significantly from the reference stage, indicating superior discriminatory power compared to FIGO stage. Note that we excluded 5 patients who received radiotherapy (RT) alone and 6 patients who received pembrolizumab from this analysis due to the small sample size. To address multicollinearity between FIGO IIIC stages and nodal status in the FIGO model, we have calculated an additional model excluding nodal status (see Supplementary Figure S2). ** indicates high and *** very high significance. # Events indicates Number of Events.

To evaluate the prognostic power of the FIGO and the ontogenetic tumor staging systems, we built two multivariable Cox regression models based on cancer-specific survival controlling for age, tumor size, histological subtype, nodal status, and treatment regime (Figure 4). While the FIGO stages showed substantially overlapping hazard ratios for the various stages, only the ontogenetic staging system demonstrated a progressive and statistically significant worsening of prognosis across all its substages. The Akaike information criterion (AIC) was 968.6 for the FIGO model and 949.9 for the ontogenetic model, indicating better performance of the ontogenetic model.

Since multicollinearity between the FIGO IIIC1 and IIIC2 stages and nodal status is a concern, we assessed this using variance inflation factors (VIFs) and the condition number (κ) of the Cox model design matrix. FIGO IIIC1, FIGO IIIC2, and nodal status showed high collinearity (VIFs: 21.8, 7.7, and 19.5), consistent with their staging dependency. The condition number (12.9) indicated moderate overall multicollinearity in the model. We therefore recalculated the model to exclude nodal status as an individual variable (Figure S2). Overall model performance, as indicated by an AIC of 970.9, was unchanged and still substantially lower than that of the oT model. To account for nodal-positive patients with local bladder/rectal infiltration (not captured in FIGO stages IIIC1 and IIIC2), we elected to leave nodal status as an individual variable in the primary model.

**Figure 5:**
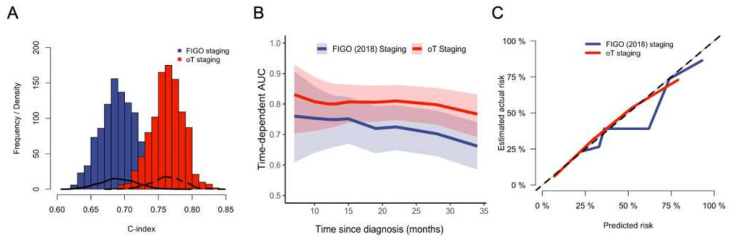
Formal comparisons of univariable Cox regression models for cancer-specific survival using the oT (red) and the FIGO (blue) staging system. (**A**) Histograms and density lines displaying C-index distribution derived from nonparametric bootstrap resampling for both models. A higher C index indicates better discriminatory power. (**B**) Time-dependent AUCs (tAUCs) for both models. At all timepoints, oT staging shows a higher AUC, indicating better prognostic power. (**C**) Calibration plot displaying predicted versus observed cancer-specific survival, along with the 45° reference line; closer agreement with the reference line indicates better calibration.

Next, we formally compared both staging systems in univariable models using Harrell’s concordance index, model calibration, and time-dependent areas under the receiver operating characteristic curve fitting as described in the statistics section (Figure 5). We found that Harrell’s concordance index was consistently higher for the ontogenetic staging model in over 1000 resampled cohorts (mean: 0.69 [95% CI: 0.62–0.72] vs. 0.75 [95% CI: 0.71–0.8] for FIGO and ontogenetic staging, respectively, Figure 5A). The mean difference in concordance index was 0.07 (95% CI: 0.04–0.13), indicating statistical significance. Furthermore, time-dependent areas under the receiver operating characteristic curve fitting revealed that the ontogenetic staging system had a higher area under the curve (AUC) for all timepoints (Figure 5B). Finally, the calibration plot depicted in Figure 5C demonstrates the superiority of the ontogenetic model in predicting cancer-specific death, with an almost excellent correlation of predicted and actual risk of cancer-related death.

**Figure 6:**
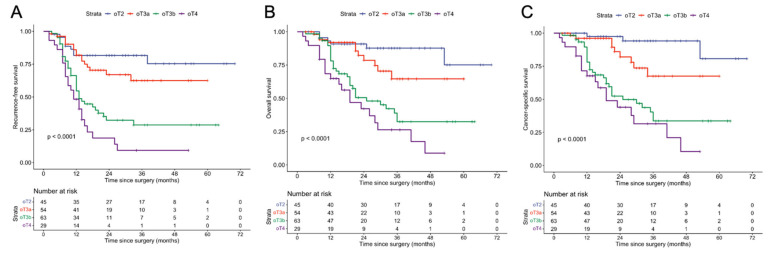
Survival for FIGO IIIC1/IIIC2: Kaplan–Meier curves depicting recurrence-free (**A**), overall (**B**), and cancer-free survival (**C**) for patients with FIGO IIIC1 and IIIC2 disease, stratified by ontogenetic tumor (oT) stage. *p*-values were calculated using the log-rank test.

As the FIGO 2018 staging system lumps almost all nodal-positive patients into the IIIC category, we investigated whether ontogenetic staging provides additional benefits within this group. We found that for all—overall survival, relapse-free survival, and cancer-specific survival—ontogenetic staging was able to stratify patients with lymph node metastases into distinct risk groups (Figure 6).

**Figure 7:**
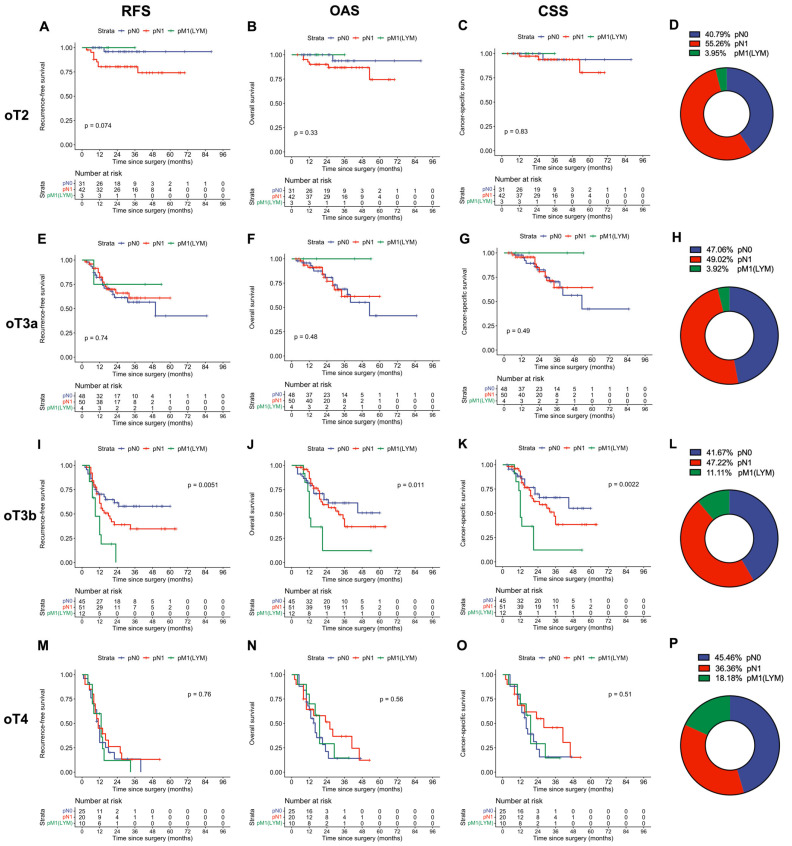
Survival outcomes for each oT stage, stratified for nodal status and lymph node metastasis region: recurrence-free (RFS), overall (OAS), and cancer-specific survival (CSS) for each ontogenetic tumor (oT) stage: oT2 (**A**–**C**), oT3a (**E**–**G**), oT3b (**I**–**K**), and oT4 (**M**–**O**). *p*-values were calculated using the log-rank test. In addition, the frequency of lymph node metastasis in the pelvic (pN1) and para-aortic (pM1(LYM)) drainage regions is indicated (**D**,**H**,**L**,**P**).

Building on these findings, we expanded our analysis for oT substages to investigate whether additional stratification by nodal status could add prognostic information, and nodal status was only a significant predictor of survival within the oT3b subset of patients (Figure 7). Interestingly, we also observed a trend in which lymph node metastases in the para-aortic region were associated with a good prognosis in early oT stages (oT2 and oT3a) but a dismal outcome in oT3b and oT4 stages.

**Figure 8:**
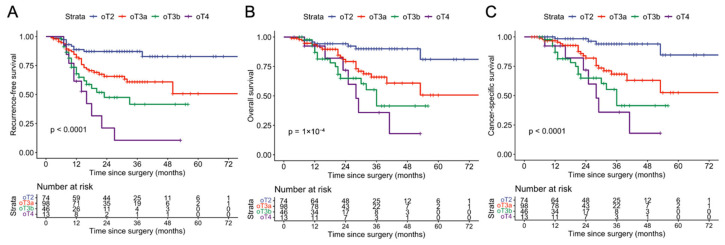
Survival for patients with parametrial infiltration: Kaplan–Meier plots representing recurrence-free (**A**), overall (**B**), and cancer-specific survival (**C**) for patients with parametrial infiltration as their maximal tumor extension (2009 FIGO IIB). *p*-values were calculated using the log-rank test. This figure highlights the prognostic utility of the oT staging system irrespective of nodal status.

Since the parametrium constitutes a complex anatomical structure with ontogenetically distinct subdomains, we investigated, in another exploratory analysis, whether the prognostication of patients with parametrial tumor infiltration, regardless of nodal status (corresponding to FIGO 2009 IIB), could be improved by ontogenetic substaging. Again, we found a significant stratification of these patients into distinct risk groups depending on ontogenetic tumor stage (Figure 8).

## 4. Discussion

In this observational retrospective cohort study, we present survival data from patients with locally advanced cervical cancer treated primarily with radiation-based regimens, comparing two staging classifications.

First, it is important to highlight a fundamental structural difference between the two staging systems. The FIGO system is based on traditional anatomical concepts combined solely with clinical and radiological findings. In contrast, the ontogenetic classification not only evaluates pelvic anatomy in greater detail but also establishes a direct link between the area of tumor involvement and tumor biology.

This new staging approach is based on the mechanisms of tissue repair and regeneration, which cancer pathologically exploits through uncontrolled cell proliferation and persistent inflammatory repair signaling. As a result, tumors behave like “wounds that do not heal.” Although cancer cell dedifferentiation is biologically related to attempts to restore tissue homeostasis, it paradoxically broadens the cells’ topoanatomical potential and promotes progressive invasion, creating a self-sustaining cycle of tissue damage [26].

Consequently, malignant progression occurs within hierarchical, developmentally defined permissive regions—known as cancer fields—which correspond to the mature derivatives of embryologic morphogenetic fields along the reverse differentiation pathway of the cell of origin. Identifying malignant cells within these anatomically mapped compartments (cancer fields) therefore determines the ontogenetic stage of the tumor.

This ability to provide clear biological discrimination between the stages of tumor progression is evidenced in a comparison of the Kaplan–Meier curves (Figure 3), revealing a more apparent prognostic distinction between ontogenetic stages than between FIGO 2018 stages. Although the analysis included only four ontogenetic subgroups and excluded lymph node status, the survival curves were markedly distinct, with minimal crossover, especially at longer follow-ups. This superior prognostic stratification was further supported by Cox regression analysis (Figure 4), which demonstrated a statistically significant, stepwise increase in cancer mortality risk with increasing ontogenetic stage. The FIGO 2018 system, on the other hand, did not establish a uniform prognostic gradient and showed inconsistent HR variation across stages, even when excluding nodal status to account for multicollinearity (Figure S2). Notably, stage IIIA and IIIB were associated with significantly worse survival than stage IIIC1, indicating a significant mismatch between tumor staging and biology. In contrast, the ontogenetic staging system provided a precise and uniform characterization of prognosis, demonstrating a steady, stepwise increase in cancer-specific mortality with each incremental stage. We confirmed these observations with additional formal testing (Figure 5).

One of the main reasons why the FIGO (2018) staging system performs so poorly is that it relies heavily on lymph node assessment. However, the prognostic significance of lymph node involvement relative to local tumor staging remains controversial in an advanced disease setting. As surgery is rarely performed in such cases, lymph node staging relies on imaging, which is often inaccurate [5]. Even PET/CT imaging, which is considered the gold standard by many, has significant limitations: its false-negative rate for pelvic nodes ranges from 24% to 27.7% [27,28], and its negative predictive value for para-aortic nodes is only 77.6% [29]. This is corroborated by the findings of Ramirez et al., who reported that 22% of patients with PET/CT-negative para-aortic nodes had histologically confirmed metastases [30]. Furthermore, as evidenced by the UTERUS 11 trial [31], even surgical staging—a potentially more accurate method—confers a benefit only in specific subgroups. In that trial, a significant improvement in oncological outcome was observed only in patients with FIGO stage IIB disease who underwent surgical retroperitoneal staging before chemoradiation.

This limitation of lymph node staging based solely on imaging may explain the weak prognostic correlation with survival observed when oT stages were stratified by lymph node status, as shown in Figure 7. Interestingly, we observed that para-aortic lymph node metastases in earlier oT stages (oT2 and oT3a) are associated with a good prognosis, while they are linked to a dismal survival outcome in later (oT3b and oT4) stages. This is in line with previously published findings indicating that (isolated) para-aortic lymph node metastases in smaller tumors with uterine corpus infiltration can be considered first-line metastases disseminating via the mesenteric drainage pathway, carrying a favorable prognosis. At the same time, para-aortic lymph node metastases in larger tumors are usually second- or third-line metastases spreading via the retroperitoneal, lumbar lymphatic drainage pathway [13]. Nevertheless, these findings should be interpreted with caution due to the limited use of PET/CT in this study, which may have introduced bias. Of note, however, in previous studies employing surgical staging, regional lymph node involvement appears to have a lesser impact on oncologic outcomes compared with ontogenetic tumor status [9,10].

Inaccurate nodal staging by imaging is probably not the only factor explaining the poor performance of the FIGO 2018 staging system. Even with perfect assessment of lymph nodes, patients with microscopic lymph node metastases would still be grouped with other patients suffering from advanced parametrial tumor involvement, and comparing local versus locoregional states in isolation, two clearly biologically distinct situations, may generate ambiguity. Yet the FIGO 2009 system (which does not consider nodal status) fails to accurately reflect prognosis, highlighting inconsistencies in the traditional anatomical view of pelvic cancer spread [32,33,34]. Key areas such as the bladder muscle, the mesorectum, and the parietal structures are often overlooked despite their prognostic significance. In contrast, our mapping of local tumor infiltration supports the anisotropic spread of cervical cancer, a finding consistent with the ontogenetic model, revealing common areas of tumor involvement that are not fully accounted for by either the FIGO 2009 or 2018 staging systems [35]. One key anatomical structure that demonstrates the impact of ontogenetic staging is the parametrium, which contains several ontogenetically distinct components (Table 1, Figure 1). Prognosis varies substantially based on which of these parametrial regions is involved, with ontogenetic stages showing distinct three-year survival rates (Figure 8). Despite this, accurate MRI-based assessment of the parametrium remains challenging, particularly for initial cases. Pathological positivity rates range from 21% to 55% in clinically staged IIB patients [36]. In this study, approximately 100 cases with early parametrial invasion were shared among different radiologists and authors for discussion, highlighting the difficulty of evaluating the parametrium solely on imaging, a common situation in an oT2 vs. oT3a scenario. It has previously been shown that the combination of clinical findings and MRI improves staging accuracy in cervical cancer [37], a potential useful tool in cases with doubtful images. Nevertheless, these factors were not included in our analysis to minimize potential bias related to the inherent subjectivity of physical examination, which may make reproducibility difficult.

An area to be explored in the future is the adaptation of modern radiotherapy strategies to target embryological compartments susceptible to neoplastic involvement, for which a clear theoretical rationale exists. Though this might necessitate adaptation of radiotherapeutic target volumes, we expect that total irradiation volumes will not change with this approach, as some areas that are currently considered high-risk regions can be spared. In addition, patients with oT2 and selected oT3a tumors may be candidates for cancer field surgery, and imaging-based staging can assist in identifying appropriate candidates for this approach [14,38].

Some limitations of our study should be discussed: First, this was a retrospective study, which has inherent limitations. Furthermore, the two participating countries were unequally represented in the study, and there was significant heterogeneity in treatment across participating centers (Table 3). Nevertheless, the fact that the prognostic power of the ontogenetic staging system was even apparent in such a heterogeneous population makes our findings more robust. Second, ontogenetic staging was based on imaging-only assignment of subtle tissue subdomains, a task usually performed in conjunction with histopathological analysis, the gold standard for tumor infiltration assessment. Third, patients with diseases limited to the cervix and superior vagina with pelvic lymph node involvement, although formally staged FIGO IIIC1, are typically treated surgically in the participating centers and were therefore excluded from this analysis, which limits the extrapolation of our findings to this group of patients.

Another relevant consideration is the generalizability of our findings to other clinical settings, especially regarding the complexity of the ontogenetic staging system, which requires specific training and practice. Furthermore, our study did not assess interobserver differences between gynecologic oncologists and radiologists, as all the imagens were reviewed by both specialties. This aspect warrants future investigation to determine the practical applicability and reproducibility of the new staging system when based solely on imaging studies.

Despite limitations and the inherent constraints of imaging modalities, this study was the first to show that the incorporation of ontogenetic anatomy based solely on imaging can enhance prognostic assessment by better aligning tumor biology with disease staging. Our findings challenge current assumptions about local spread in cervical cancer and suggest the need to reassess staging methods more broadly.

## 5. Conclusions

In conclusion, for locally advanced cervical cancer, ontogenetic staging can be effectively applied using imaging alone and was superior to the FIGO 2018 classification in this study. Although its adoption requires a paradigm shift, the ontogenetic model of cancer spread—and its associated staging system—has the potential to improve management of locally advanced cervical cancer.

## Figures and Tables

**Table 1:** Guideline for pelvic MRI evaluation of ontogenetic tumor stage.

Ontogenetic Tumor (oT) Stage	Description	Landmarks on MRI
oT1	The tumor is limited to the cervical stroma.	The pelvic MRI in the T2 mode shows an uninterrupted dark cervical border zone in both transverse and sagittal planes and no evidence of tumor invasion of the uterine corpus.
oT2	The tumor is limited to the Müllerian compartment (includes cases of initial parametrial involvement limited to the paracervix, without fatty tissue/mesometrium invasion).	Pelvic MRI in the T2 mode can show an interrupted dark cervical border zone in both transverse and sagittal planes, without signs of invasion on urogenital mesentery components (uterine artery with fatty tissue, uterosacral ligaments, bladder mesentery, and inferior hypogastric plexus branches) or bladder adventitia. Uterine corpus invasion. Proximal vagina invasion.
oT3a	The cancer infiltrates any anatomical compartment derived from the Müllerian duct morphogenetic field.	Involvement of any of the following structures: Bladder adventitia. Proximal bladder mesenteries. Paraurethral tissues. Vascular and ligamentous mesometria and mesocolpium. Genital serosa. Inferior hypogastric plexus.
oT3b	The cancer infiltrates any compartment matured from the primordial genital tract morphogenetic field.	MRI shows extracervical tumor spread up to the border of the parietal (somatic) retro-, subperitoneum, and peritoneum; endopelvic fascia; and mesorectum, but there is no evidence of infiltration of these tissues. Unilateral or bilateral ureteral dilatation is often present but not obligatory. Tumor involvement of the urethral and bladder wall with or without infiltration of the mucosal layer is often obvious with MRI. Involvement of any of the following structures: Bladder muscle or mucosa. Bladder peritoneum. Distal urogenital mesenteries and umbilical arteries. Endopelvic fascia. Ovaries. Paramesenteric retroperitoneum, subperitoneum, and/or peritoneum with the pararectal fascia.
oT4	The most advanced ontogenetic local tumor stage is evident if the cancer infiltrates tissues derived from the mesonephric system morphogenetic field, in addition to the oT3b cancer field. oT4 tumors can infiltrate any pelvic and abdominal tissue, except the spinal column and adjacent autochthonous musculature.	Involvement of any of the following structures: Mesorectum. Rectum with or without mucosal involvement. Mesureter and ureter. Any pelvic and abdominal tissue, except the spinal column and adjacent autochthonous musculature.

**Table 2:** Corresponding nomenclatures between both staging systems.

Ontogenetic Anatomical Structure	Corresponding Conventional Anatomic Structure
Urogenital mesentery	Structure composed of uterine arteries and veins with their surrounding fatty tissue, bladder mesenteries, inferior hypogastric plexus, and vaginal vessels with their adjacent fatty tissue. Corresponds to the parametrium with its fatty tissue portion.
Distal urogenital mesentery	Distal parametrium portion up to the pelvic side wall, subperitoneum and endopelvic fascia. Usually corresponds to FIGO IIIB.
Vascular mesometrium	Uterine arteries and veins with their surrounding fatty tissue.
Ligamentous mesometrium	Uterosacral ligaments.
Mesocolpium	Sacrovaginal ligaments.
Parietal structures	Pelvic muscles, sacral roots, sciatic nerve, bone, and major vessels.

**Table 3:** Patients’ characteristics by treatment center.

Characteristic	Brazil *N* = 274 ^1^	Russia *N* = 67 ^1^	*p*-Value ^2^
Patient age (years)	48 (38, 58)	52 (45, 65)	0.003
FIGO (2018) stage			0.046
IIB	65.0 (23.7%)	28.0 (41.8%)	
IIIA	5.0 (1.8%)	1.0 (1.5%)	
IIIB	23.0 (8.4%)	8.0 (11.9%)	
IIIC1	139.0 (50.7%)	24.0 (35.8%)	
IIIC2	24.0 (8.8%)	4.0 (6.0%)	
IVA	18.0 (6.6%)	2.0 (3.0%)	
Ontogenetic tumor (oT) stage			0.14
oT2	60.0 (21.9%)	16.0 (23.9%)	
oT3a	77.0 (28.1%)	25.0 (37.3%)	
oT3b	87.0 (31.8%)	21.0 (31.3%)	
oT4	50.0 (18.2%)	5.0 (7.5%)	
Lymph node metastasis	176.0 (64.2%)	28.0 (41.8%)	<0.001
Tumor size (mm)	53 (42, 65)	49 (42, 59)	0.2
Histologic subtype			0.11
Adenocarcinoma	27.0 (9.9%)	10.0 (14.9%)	
Adenosquamous carcinoma	2.0 (0.7%)	2.0 (3.0%)	
Squamous cell carcinoma	245.0 (89.4%)	55.0 (82.1%)	
Local staging (pelvis)			
Pelvic MRI	274 (100%)	67 (100%)	
Retroperitoneum, upper abdomen and chest evaluation			
CT scans	274 (100%)	67 (100%)	
PET/CT	10 (3.6%)	6 (8.9%)	
Treatment			<0.001
Concurrent chemoradiotherapy	223.0 (81.4%)	35.0 (52.2%)	
Induction chemotherapy + concurrent chemoradiotherapy (same as INTERLACE trial regimen)	42.0 (15.3%)	30.0 (44.8%)	
Pembrolizumab + concurrent chemoradiotherapy (same as Keynote-A18 trial regimen)	4.0 (1.5%)	2.0 (3.0%)	
Radiotherapy only	5.0 (1.8%)	0.0 (0.0%)	
Type of radiotherapy tecnique			
EBRT (IMRT) 40–50 Gy + brachytherapy 28–40 Gy	70 (25.5%)	67 (100%)	
EBRT (3D CRT) 40–50 Gy + brachytherapy 28–40 Gy	204 (74.4%)	0	
Extended-field EBRT (IMRT)	4 (1.4%)	4.0 (6.0%)	
Extended-field EBRT (3D CRT)	20.0 (7.3%)	0	
Recurrence type			<0.001
distant	14.0 (5.1%)	3.0 (4.5%)	
local	76.0 (27.7%)	4.0 (6.0%)	
local + distant	18.0 (6.6%)	4.0 (6.0%)	
no recurrence	166.0 (60.6%)	56.0 (83.6%)	

^1^ Median (Q1, Q3); n (%); ^2^ Wilcoxon rank sum test; Fisher’s exact test; Pearson’s Chi-squared test.

## Data Availability

Raw data can be requested from the corresponding author.

## Supplementary Materials

The following supporting information can be downloaded at: https://www.mdpi.com/article/10.3390/cancers18040689/s1, Table S1: Types of relapses and treatment for relapses; Table S2: Other causes of death (unrelated to cancer); Table S3: Ontogenetic stage of and local involvement on pelvis; Table S4: Numeric survival data (from Figure 3); Table S5: oT-stage distribution within each FIGO (2018) stage; Figure S1: Kaplan–Meier curves depicting recurrence-free (A), overall (B), and cancer-specific (C) survival of patients with FIGO IIIC1 disease, stratified for ontogenetic tumor stage; Figure S2: Forestplot depicting the results of a multivariable Cox regression model using FIGO (2018) stage as the primary covariate and controlling for age, tumor size, and histological subtype.
