# Supplementary material for: FIGO 2018 Versus Ontogenetic Staging for Locally Advanced Cervical Cancer: An International Multicenter Cohort Study Comparing the Two Classifications and Their Prognostic Implications
Source: Cancers (Basel). 2026 Feb 19;18(4):689. doi: 10.3390/cancers18040689 (PMC12938924; doi:10.3390/cancers18040689)
Supplement: Supplementary file 1 [file cancers-18-00689-s001.zip › cancers-4119443-supplementary.pdf]

Supplementary Materials:

Table S1: Types of relapses and treatment for relapses. Table S2: Other causes of death (unrelated to cancer). Table S3: Ontogenetic stage of and local involvement on pelvis. Table S4: Numeric survival data (from Fig. 2). Table S5: Patient characteristics by treatment center.

Table S1: Types of relapses and treatment for relapses.

**Type of relapse (%)**	**Treatment on relapse (%)**
Locoregional* only (23.9)	Exclusive palliation (42.8), chemotherapy (41.6), surgery (11.9), observation (2.3), and radiotherapy (1.1)
Distant only (5.3)	Chemotherapy (42.0), exclusive palliation (31.5), surgery (15.7), radiotherapy (5.2%), and observation (5.2%)
Locoregional* + distant (6.7)	Chemotherapy (62.5) and exclusive palliation (37.5)

(*) Locoregional: includes retroperitoneal lymph nodes.

Table S2: Other causes of death (unrelated to cancer).

**Cause of death (unrelated to cancer)**	**Number of cases**
Pulmonary sepsis	4
Intestinal obstruction/late actinic complications (> 6 months after the end of treatment)	4
Unknown	1

Table S3: Ontogenetic stage of and local involvement on pelvis.

**Ontogenetic tumor stage (oT)**	**Most common anatomical areas of involvement defining ontogenetic tumor stage (%)**
oT2	Paracervix (94.8), superior vagina (61.5), and paracolpium (59.1)

oT3a	Proximal urogenital mesentery* (73.2) and ligamentous mesometrium or mesocolpium (50.0)
oT3b	Bladder muscle (85.3) and distal urogenital mesentery (59.6)
oT4	Mesorectum (88.3), rectum serosa (33.3), and parietal structures (15.0)

(*) Urogenital mesentery: structure composed of uterine arteries and vein with their surrounding fatty tissue, bladder mesenteries, inferior hypogastric plexus, and vaginal vessels with their adjacent fatty tissue).

Table S4: Numeric survival data (from Fig. 3).

<table>
<tr><th colspan="2">Stage</th><th>n</th><th>3-year RFS</th><th>3-year OAS</th><th>3-year CSS</th></tr>
<tr><td colspan="2">All stages</td><td>341</td><td>51.7 (46.0 - 58.2)</td><td>57.7 (51.5 - 64.5)</td><td>60.8 (54.5 - 67.8)</td></tr>
<tr><td rowspan="6">FIGO 2018</td><td>IIB</td><td>93</td><td>72.9 (63.0 - 84.4)</td><td>75.8 (65.5 - 87.8)</td><td>76.6 (66.3 - 88.6)</td></tr>
<tr><td>IIIA</td><td>6</td><td>44.4 (16.7 - 100)</td><td>41.7 (14.7 - 100)</td><td>41.7 (14.7 - 100)</td></tr>
<tr><td>IIIB</td><td>32</td><td>49.7 (33.0 - 75.0)</td><td>62.0 (45.3 - 84.7)</td><td>72.2 (54.9 - 94.9)</td></tr>
<tr><td>IIIC1</td><td>162</td><td>52.4 (44.3 - 62.0)</td><td>58.1 (49.2 - 68.8)</td><td>62.2 (53.0 - 73.1)</td></tr>
<tr><td>IIIC2</td><td>28</td><td>24.4 (11.0 - 53.9)</td><td>26.3 (12.0 - 57.8)</td><td>26.3 (12.0 - 57.8)</td></tr>
<tr><td>IVA</td><td>20</td><td>0</td><td>12.4 (3.5 - 44.4)</td><td>13.6 (3.8 - 48.0)</td></tr>
<tr><td rowspan="4">Ontogenetic tumor stage</td><td>2</td><td>76</td><td>87.5(80.2 - 95.5)</td><td>90.3 (83.0 - 98.2)</td><td>94.1 (87.8 - 100)</td></tr>
<tr><td>3a</td><td>102</td><td>59.8 (49.3 - 72.5)</td><td>67.3 (56.0 - 80.7)</td><td>69.5 (58.2 - 83.1)</td></tr>
<tr><td>3b</td><td>108</td><td>41.2 (31.6 - 53.4)</td><td>43.9 (33.6 - 57.4)</td><td>46.2 (35.5 - 60.1)</td></tr>
<tr><td>4</td><td>55</td><td>10.0 (3.9 - 26.0)</td><td>23.2 (13.1 - 40.9)</td><td>27.3 (16.1 - 46.5)</td></tr>
</table>

Table S5: oT-stage distribution within each FIGO (2018) stage.

	**oT2** N = 76[1]	**oT3a** N = 102[1]	**oT3b** N = 108[1]	**oT4** N = 55[1]
**FIGO (2018) stage**				
IIB	30 (39%)	46 (45%)	15 (14%)	2 (4%)
IIIA	1 (1%)	1 (1%)	3 (3%)	1 (2%)
IIIB	0 (0%)	1 (1%)	21 (19%)	9 (16%)
IIIC1	42 (55%)	50 (49%)	51 (47%)	20 (36%)
IIIC2	3 (4%)	4 (4%)	12 (11%)	9 (16%)
IVA	0 (0%)	0 (0%)	6 (6%)	14 (25%)

[1]n (%).

Figure S1:

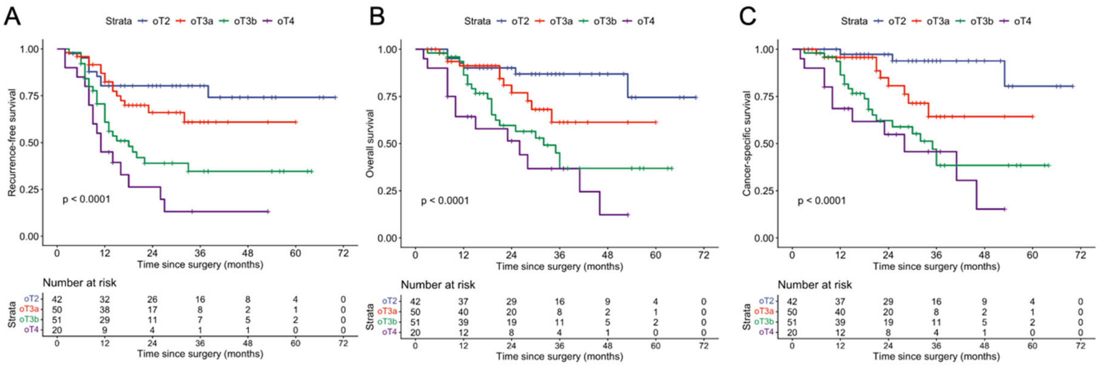

Kaplan–Meier curves depicting recurrence-free (A), overall (B), and cancer-specific (C) survival of patients with FIGO IIIC1 disease, stratified for ontogenetic tumor stage.

Figure S2:

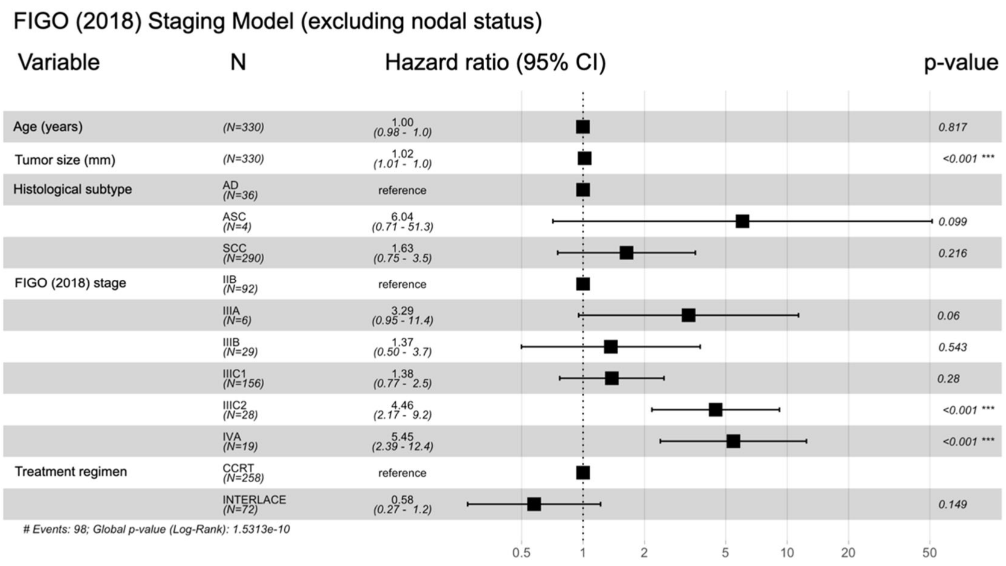

Forestplot depicting the results of a multivariable Cox regression model using FIGO (2018) stage as the primary covariate and controlling for age, tumor size, and histological subtype. Contrary to the model depicted in Figure 4 in the main text, this model excludes nodal status as an individual variable to address multicollinearity, as discussed in the main text. The overall model performance remains similar, as indicated by an AIC value of 970.9 (compared to 968.6 in the model including nodal status).
